# The tumor suppressive role of NUMB isoform 1 in esophageal squamous cell carcinoma

**DOI:** 10.18632/oncotarget.2136

**Published:** 2014-06-26

**Authors:** Junmou Hong, Zhenguo Liu, Hua Zhu, Xin Zhang, Yongju Liang, Shiyuan Yao, Fang Wang, Xiaoyun Xie, Bo Zhang, Tao Tan, Liwu Fu, Jing Nie, Chao Cheng

**Affiliations:** ^1^ Department of Thoracic Surgery, The First Affiliated Hospital of Sun Yat-sen University, Guangzhou, People's Republic of China; ^2^ Department of Surgery, Davis Heart and Lung Research Institute, The Ohio State University Wexner Medical Center, Columbus, OH,USA; ^3^ State Key Laboratory of Oncology in South China, Cancer Center, Sun Yat-sen University, Guangzhou, People's Republic of China; ^4^ Division of Geriatrics, Tongji Hospital, Tongji University, School of Medicine, Shanghai, People's Republic of China; ^5^ Division of Nephrology, Nanfang Hospital, Guangzhou, Guangdong, People's Republic of China

**Keywords:** esophageal squamous cell carcinoma, ESCC, NUMB isoform 1, Aurora-A, G2/M arrest

## Abstract

Esophageal quamous cell carcinoma (ESCC) is the predominant histological type of esophageal carcinoma in Asian populations. To date, few biomarkers have been identified for ESCC. In present study, we found a tumor suppressor, NUMB isoform 1 (NUMB-1), as a promising prognostic biomarker for patients with ESCC. NUMB-1 mRNA was downregulated in 66.7% of primary ESCC tissues when compared with matched adjacent non-tumor tissues. The low expression of NUMB-1 was significantly associated with high tumor recurrence (*p*=0.029) and poor post-operative overall survival (*p*=0.016). To further explore the underlying mechanisms by which NUMB-1 regulates ESCC, we demonstrated that ectopic expression of NUMB-1 inhibited cell proliferation through inducing G2/M phase arrest, which was accompanied by an increase in p21 and cyclin B1-cdc2 levels. However, it had no impact on apoptosis of ESCC cells. In addition, overexpression of NUMB-1 prevented epithelial-mesenchymal transition, inhibited invasion of ESCC cells and NOTCH pathway, suppressed Aurora-A activity by preventing phosphorylation of Aurora-A at T288 which resulted in cell cycle arrest. Taken together, our findings suggested NUMB-1 functions as a tumor-suppressor and serves as a prognositc biomarker for ESCC patients; thus, NUMB-1 may be a potential novel therapeutic target for treatment of ESCC.

## INTRODUCTION

Esophageal carcinoma is the seventh most common cancer and the fifth leading cause of cancer-related death worldwide [1]. Esophageal squamous cell carcinoma (ESCC) is the predominant form and accounts for greater than 90% of esophageal cancer cases in China [2, 3]. Despite recent advances in multimodality therapies, the 5-year survival rate for ESCC is still below 30% [4]. ESCC is believed to develop due to a variety of risk factors including the accumulation of genetic mutations, tobacco and alcohol consumption, obesity, and diet [5]. To date, there are few specific biomarkers available for diagnostic use and in the development of targeted therapies against this devastating disease. Hence, characterization of molecular markers involved in the initiation and progression of ESCC is essential.

NUMB protein was first identified and studied in *Drosophila* [6] where it was believed to modulate asymmetric self-renewal and progenitor cell fate determination during cell division [7-9], endocytosis, cell adhesion, and migration [10, 11]. The relationship between NUMB and tumorigenesis was discovered in recent studies [12, 13] and accumulating evidence suggests a potential tumor suppressor role for NUMB, including stabilization of p53 [14, 15] and inhibition of Notch signaling [16, 17]. Indeed, these proposed tumor suppressive functions of NUMB are consistent with previous studies conducted in breast cancer [18], non-small cell lung cancer (NSCLC) [19], and salivary gland carcinomas [20] in which tumors exhibited reduced expression of NUMB. On the other hand, overexpression of NUMB was found in astrocytomas [21] and cervical squamous carcinoma cells [22], suggesting that NUMB also possesses oncogenic potential. In mammals, it has been shown that NUMB encodes six alternatively spliced transcripts (NUMB isoforms 1-6) [23]. Previous studies have reported that NUMB-2 and NUMB-4 promote glioma cell growth *in vivo* [24] while NUMB-5 and NUMB-6 promote tumor formation and development [25]. Currently, little is known about the role of NUMB-1 and NUMB-3 in tumorigenesis.

These findings raise the possibility that the role of NUMB in tumorigenesis may be tumor specific and isoform specific. Few studies, up to date, have examined the involvement of NUMB and its isoforms in the pathogenesis of ESCC. Herein, we report low expression of NUMB-1 in tumor tissues and the association of NUMB-1 downregulation with poor prognosis in ESCC patients. Furthermore, we demonstrate a multifunctional role for NUMB-1 in the inhibition of cell proliferation, EMT as well as in cell cycle G2/M arrest through interacting and de-phosphorylating Aurora-A and inhibiting NOTCH pathway. These findings suggest that NUMB-1 is a novel, putative tumor suppressor and a therapeutic target of ESCC.

## RESULTS

### NUMB-1 mRNA level is down-regulated in ESCC tissues and correlates with poor prognosis in patients

**Fig.1:**
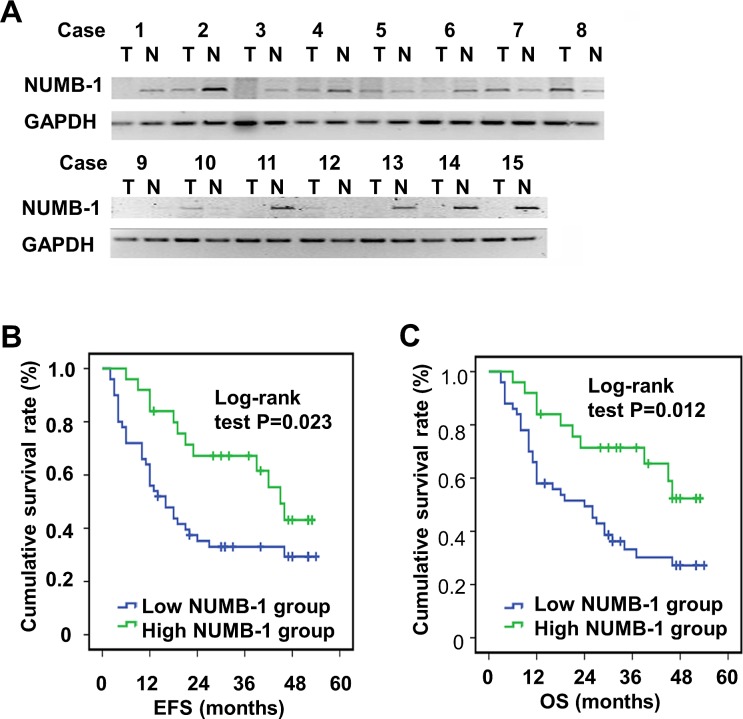
The mRNA level of NUMB-1 is decreased in ESCC tissues and correlates with poor prognosis in patients Semi-quantitive PCR was performed to detect NUMB-1 mRNA levels in ESCC tissues and adjacent normal tissues resected from 75 patients. (A) A representative image showing decreased NUMB-1 mRNA levels in 10 out of 15 pairs of tissue. (B) Kaplan–Meier analysis showed that patients in the low NUMB-1 group (n=50) experienced a significantly shorter EFS (median EFS: 16 months vs 45 months, p=0.023, log-rank test) and overall survival (OS) (median OS: 24 months vs 49 months, p=0.012, log-rank test) than patients in the high NUMB-1 group (n=25).

We first examined NUMB-1 mRNA levels in 75 pairs of ESCC tumor tissues along with their adjacent non-cancerous tissue. As shown in Fig. 1A, representative PCR result for 15 pairs of tissues show that NUMB-1 mRNA expression in ESCC tumor tissues was decreased in 10 out of 15 pairs, increased in 5 out of 15 pairs when comparing with ajacent non-cancerous tissue. According to the NUMB-1 level in tumor tissues, compared to the ajacent non-cancerous tissue, patients were devided into the low NUMB-1 group (n=50) and high NUMB-1 group (n=25). Correlating NUMB-1 expression to patient prognosis, we discovered that the low NUMB-1 group experienced a significantly shorter event-free survival (EFS) (median EFS: 16 months vs 45 months, p=0.023, log-rank test) and overall survival (OS) (median OS: 24 months vs 49 months, p=0.012, log-rank test) compared with patients in the high NUMB-1 group (Fig. 1 B, C). We investigated the association between NUMB-1 expression and the characteristics of the patients in greater detail. Results showed that downregulation of NUMB-1 was significantly linked to a more advanced tumor stage (Pearson's χ^2^ test, *p*=0.048) but not to age, gender, lymph node metastasis, T stage, or tumor differentiation (Table 1). Through univariate analysis, we found that advanced tumor stage (*p*=0.012, HR: 2.121, 95%CI: 1.180-3.813), T stage (*p*=0.042, HR: 2.132, 95%CI: 1.027-4.425), and a decrease in NUMB-1 expression (*p*=0.029, HR: 2.088, 95%CI: 1.078-4.045) were all significant predictors of recurrence in ESCC patients. With regards to long-term survival, advanced tumor stage (*p*=0.002, HR: 2.619, 95%CI: 1.424-4.82), lymphatic metastasis (p=0.022, HR: 2.011, 95%CI: 1.108-3.650), T stage (*p*=0.033, HR: 2.311, 95%CI: 1.072-4.984), and a decrease in NUMB-1 expression (*p*=0.016, HR: 2.39, 95%CI: 1.180-4.860) all significantly predicted decreased overall 5-year survival (Table 2). However, the only predictor of decreased EFS (*p*=0.037, HR: 2.142, 95%CI: 1.047-4.381) and OS (*p*=0.03, HR: 2.337, 95%CI: 1.08-5.03), by multivariate analysis, was NUMB-1 downregulation (Table 3).

**Table 1:** Association between Numb-1 mRNA expression and clinical characteristics in ESCC

Variables	High Numb-1 group (n=25)	Low Numb-1 group (n=50)	*p*-value*
Age	61.8±9.78	58.3±10.2	0.165
Sex			0.375
Male	18	41	
Female	7	9	
Grade			0.200†
G1	7	7	
G2	10	30	
G3	8	13	
TNM classification			
pT			0.585
T1/T2	8	13	
T3/T4	17	37	
pN			0.069
N0	18	25	
N1-2	7	25	
Stage			0.048
I/II	18	24	
III/IV	7	26	

* Chi-square test

† Wilcoxon test. ESCC, Esophageal squamous cell carcinoma.

**Table 2:** Univariate analysis of the clinical factors influencing the prognosis of the ESCC patients

Variables	event-free survival			overall-survival		
	Risk ratio	95%CI	p-value*	Risk ratio	95%CI	p-value*
Age			0.780			0.755
≤60	1	1		1	1	
>60	0.92	0.516-1.642		1.10	0.608-1.987	
Sex			0.201			0.147
Female	1	1		1	1	
male	1.67	0.756-3.787		1.89	0.800-4.48	
Grade			0.621			0.600
G1	1	1		1	1	
G2	1.113	0.505-2.45		1.01	0.447-2.27	
G3	0.794	0.403-1.561		0.737	0.37-1.46	
TNM						
pT			0.042			0.033
T1/T2	1	1		1	1	
T3/T4	2.132	1.027-4.425		2.311	1.072-4.984	
N			0.096			0.022
N0	1	1		1	1	
N1-2	1.637	0.916-2.928		2.011	1.108-3.650	
Stage			0.012			0.002
I/II	1	1		1	1	
III/IV	2.121	1.180-3.813		2.619	1.424-4.82	
Numb1			0.029			0.016
high	1	1		1	1	
Low	2.088	1.078-4.045		2.39	1.18-4.86	

* Univariate Cox regression analysis

**Table 3:** Multivariate analysis of factors that influence prognosis of ESCC patients

Variables	event-free survival			overall-survival		
	Risk ratio	95%CI	p-value*	Risk ratio	95%CI	p-value*
Age			0.819			0.980
≤60	1	1		1	1	
>60	0.820	0.440-1.510		1.007	0.540-1.880	
Sex			0.365			0.34
Female	1	1		1	1	
Male	1.480	0.634-3.451		1.54	0.630-3.770	
Grade			0.246			0.288
G1	1	1		1	1	
G2	0.814	0.362-1.827		1.190	0.520-2.700	
G3	0.528	0.241-1.160		0.664	0.310-1.360	
TNM						
pT			0.211			0.197
T1/T2	1	1		1	1	
T3/T4	1.650	0.754-3.626		1.732	0.752-3.99	
pN			0.460			0.570
N0	1	1		1	1	
N1-2	0.680	0.249-1.890		0.740	0.26-2.090	
Stage			0.140			0.112
I/II	1	1		1	1	
III/IV	2.15	0.780-5.960		2.350	0.818-6.740	
Numb1			0.037			0.030
high	1	1		1	1	
Low	2.142	1.047-4.381		2.337	1.080-5.030	

* Multivariate Cox regression analysis

### NUMB-1 inhibits migration and invasion of KYSE150 cells by preventing EMT

**Fig.2:**
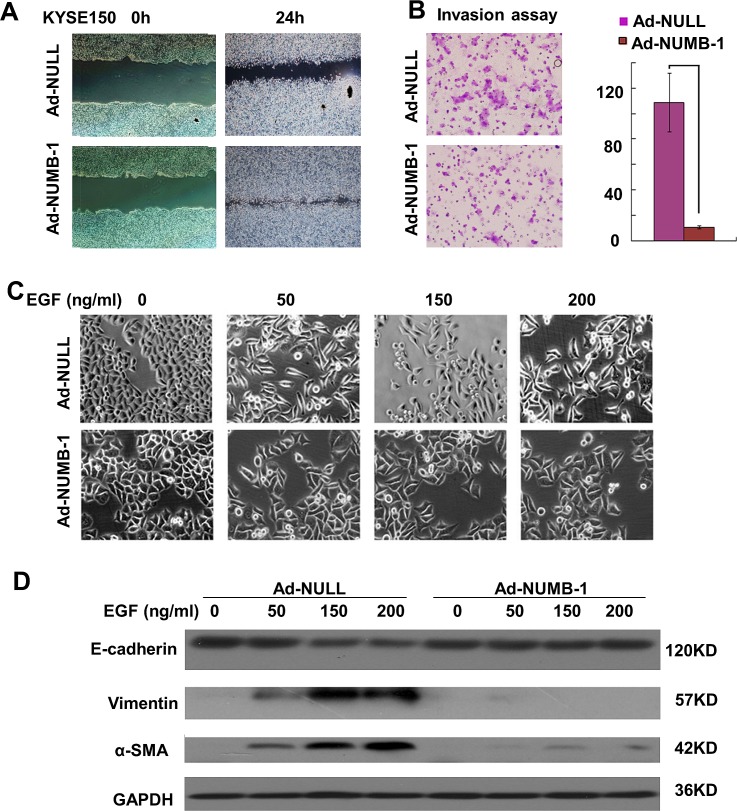
Overexpression of NUMB-1 in KYSE150 cells inhibits cell motility and EMT induced by EGF (A) A wound was introduced in sub-confluent cultures of KYSE150 cells that either overexpressed NUMB-1 (Ad-NUMB-1) or a control vector (Ad-Null). Wound closure was monitored at the indicated time points, and a representative image from three independent experiments is shown (original magnification, 200×). (B) Invasiveness of the cells was analyzed in a Boyden chamber that was either coated with Matrigel or left uncoated. Cells that invaded through the filter and adhered to the lower surface of the filter were stained and analyzed by microscopy. Results are shown as mean ± SD (* *p*<0.01). (C) The cellular morphology of control KYSE150 cells and NUMB-1-overexpressors, cultured on plastic and treated with various concentrations of EGF for 48 h after a 24h starvation period, was observed by phase contrast microscopy. The control KYSE150 cells (top panel) exhibit a spindle-like, fibroblastic morphology, while NUMB-1-overexpressing cells (bottom panel) retain a cobblestone-like appearance even upon treatment with high doses of EGF. Representative images were acquired at the time points indicated in the top left corner of each image (original magnification, 400×). (D) The expression of E-cadherin, Vimentin, and α-SMA upon EGF treatment in control cells and NUMB-1 overexpressing KYSE150 cells was analyzed by Western blotting.

Given the association between NUMB-1 expression and tumor stage, we hypothesize that NUMB-1 plays an important role in tumor growth and metastasis. To address this issue, ESCC cell line KYSE150 was infected with adenovirus encoding HA-tagged NUMB-1 (pAd-HA-NUMB-1). Migration and invasion of KYSE150 cells were dramatically decreased in NUMB-1 overexpressed cells as assessed by the scratch wound healing assay and Boyden chamber invasion assay, respectively (Fig. 2A and 2B, * *p*<0.01). We also discovered that NUMB-1 overexpression prevented EGF-induced morphological changes (i.e. a switch from cobblestone to spindle-like and fibroblastic morphology) (Fig. 2C). Furthermore, immunoblot analysis revealed that NUMB-1 overexpression attenuated EGF-induced E-cadherin suppresion and α-smooth muscle actin (α-SMA) and, vimentin induction in KYSE150 cells. (Fig. 2D).

### Overexpression of NUMB-1 suppresses growth of KYSE150 cells both *in vitro* and *in vivo*

**Fig.3:**
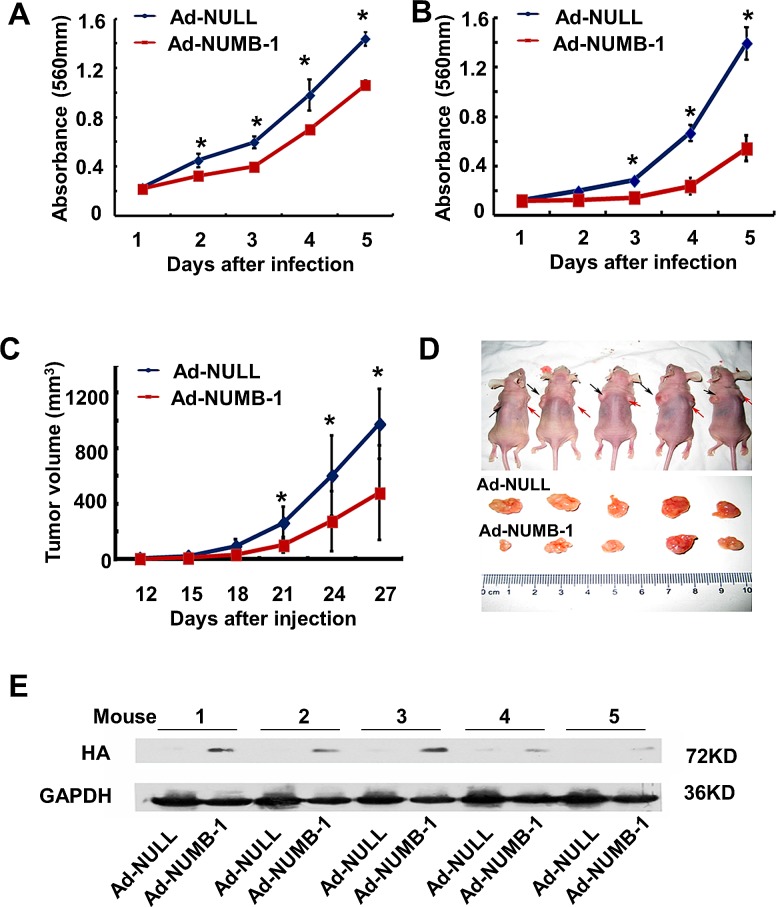
Overexpression of NUMB-1 significantly suppresses ESCC cell growth both *in vitro* and *in vivo* (A, B) MTT assay was performed in KYSE150 (A) and KYSE30 (B) cells that either overexpressed NUMB-1 or the control vector. NUMB-1 overexpression in KYSE150 cells and KYSE30 cells suppressed their growth rate as compared to the control group in the respective cell lines (* *p*<0.01). (C) *In vivo* tumor growth assay was carried out to investigate the influence of NUMB-1 on the ability of KYSE150 cells to form tumors in mice. Summary of Ad-Null and Ad-NUMB-1 tumor growth curves in nude mice showing the average tumor volume expressed as mean ± SD in inoculated sites (n=5) for each group (* *p*<0.05). (D) Mice were sacrificed 4 weeks after injection;black arrows indicate Ad-Null injection sites while red arrows indicate Ad-NUMB-1 injection sites. The tumors excised from these mice are shown below. (E) Western blot for expression of HA tag was performed to verify HA-NUMB-1 overexpression in the tumor samples.

MTT assay revealed that NUMB-1 overexpression suppressed cell proliferation in both KYSE150 and KYSE30 cells compared with cells infected with control virus (Fig. 3A, B, * *p*<0.01). We further studied the effect of NUMB-1 on ESCC tumor formation in nude mice. Overexpression of HA-NUMB-1 in KYSE150 cells inhibited its ability to grow in mice as measured by the tumor growth curve (Fig. 3C, * *p*<0.05). The tumors excised at the end of the study are shown (Fig. 3D), and the weight of xenografts in NUMB-1 overexpression group was lower than that in control group, although the difference was not significant (0.173±0.103g in NUMB-1 vs. 0.293±0.092g in control, p=0.089). Numb-1 overexpression was confirmed by western blot using antibody against- HA (Fig. 3E).

### Overexpression of NUMB-1 induces ESCC cell cycle arrest in G2/M phase

**Fig.4:**
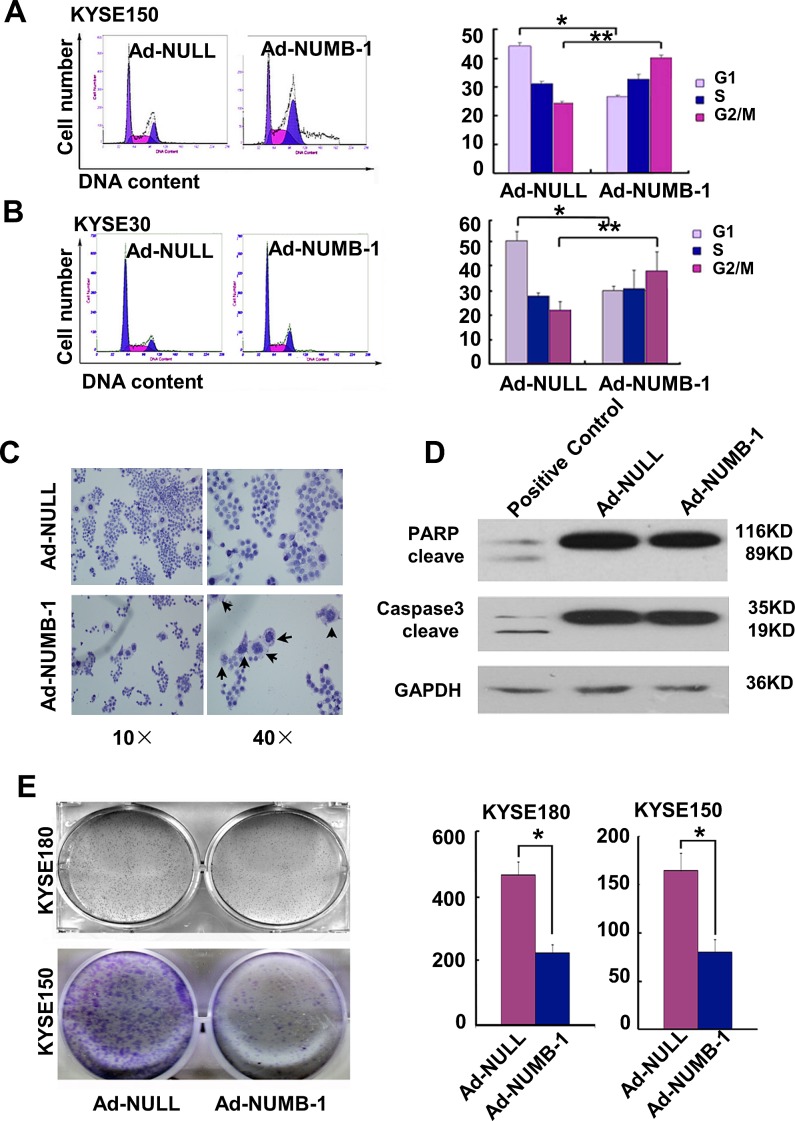
Overexpression of NUMB-1 suppresses KYSE150 cell growth by arresting cells in M phase (A) Cell cycle analysis by flow cytometry was performed to determine the change in cell cycle distribution of KYSE150 NUMB-1 overexpressors compared to KYSE150 control cells. The percentage of KYSE150 cells overexpressing NUMB-1 found in the G2/M phase (40.04±1.07%) was significantly higher than the percentage of control KYSE150 cells (24.34±0.57%). (B) A similar result was observed in KYSE30 cells with the G2/M ratio increasing from 22±3.6% in control cells to 38±7.81% in NUMB-1 overexpressors. (C) Representative images of H&E staining show an increase in polyploidy (indicated by the arrows) induced by overexpressing NUMB-1 in KYSE150 cells. (D) Western blot for caspase-3 and PARP expression demonstrate that Numb-1 could not induce caspase-3 and PARP cleavage in KYSE150 cells. The positive control was HL60 cells treated with 10 μM DDP. (E) Overexpression of NUMB-1 significantly suppressed foci formation in both KYSE150 (164±18.1 vs 80.3±12.8) and KYSE180 cells (465±42 vs 223±26) compared to control-infected cells.

To explore the mechanism underlying growth inhibition by NUMB-1, we performed flow cytometry on KYSE150 and KYSE30 cells to calculate their distribution across the different phases of the cell cycle. As shown in Fig. 4A-B, NUMB-1 overexpression induced G2/M arrest in both KYSE150 cells (increased from 24.34±0.57% to 40.04±1.07%) and KYSE30 cells (increased from 22±3.6% to 38±7.81%). KYSE150 cells overexpressing NUMB-1 exhibited a dramatic increase in polyploidy, as revealed by H&E staining (Fig. 4C), which was corroborated by the tail observed in the flow cytometric analysis of cell cycle distribution. Furthermore, the inhibition of cell cycle arrest by NUMB-1 overexpression was observed dynamically by the time-lapse imaging technology(supplemental Fig S1). However, no obvious sub-G1 peak, indicative of apoptotic cells, was detected by flow cytometry in either cell line. These data suggest the suppression of growth by NUMB-1 overexpression was not related to apoptosis, which was further confirmed by the absence of Caspase-3 and PARP cleavage (Fig. 4D). The colonial genic assays were done by using KYSE30, KYSE150 and KYSE180 cell lines. After overexpression of NUMB-1 by adenoviral gene delivery, KYSE30 was unable to grow colonies, indicating a stronger tumor suppress effect of NUMB-1. Here we demonstrated that NUMB-1 overexpression significantly suppressed *in vitro* foci formation in both KYSE180 cells (465±42 in control vs 223±26 in NUMB-1, *p*<0.05) and KYSE150 cells (164±18.1 in control vs 80.3±12.8 in NUMB-1, *p*<0.05)(Fig. 4E), suggesting that NUMB-1 overexpression could inhibit ESCC cell proliferation.

### NUMB-1 inhibits NOTCH pathway and arrests ESCC cells in M phase by interacting with Aurora-A and suppressing its activity

**Fig.5:**
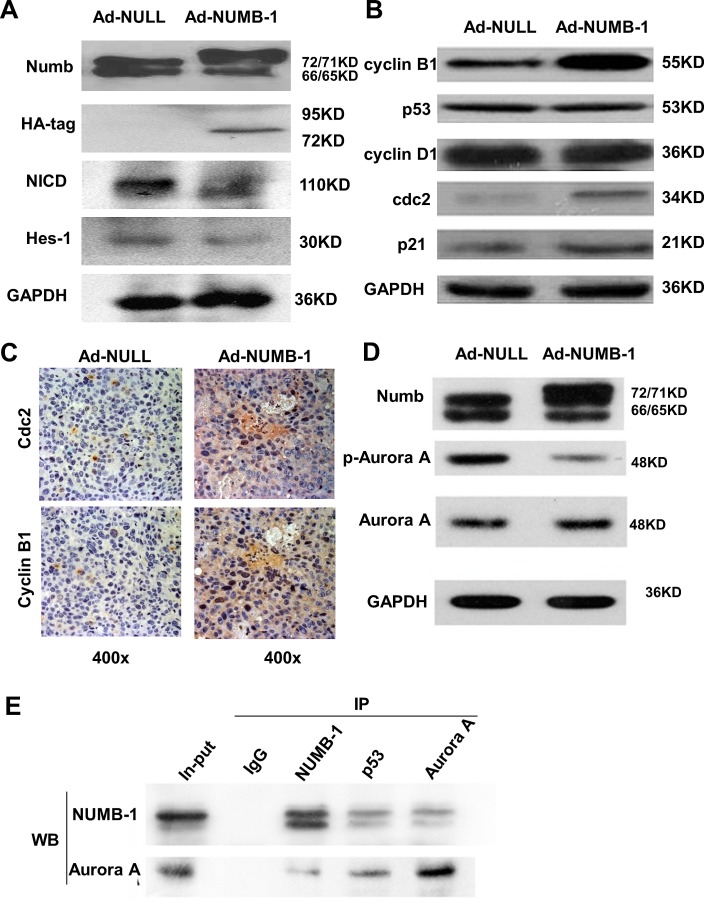
NUMB-1 induces ESCC cell cycle arrest in M phase by interacting with Aurora-A and suppressing its activity (A) Overexpression of NUMB-1 inhibits the Notch pathway. Immunoblots showed overexpression of Numb isoform 1 in KYSE150 downregulated the NICD as well as its downstream protein Hes-1. NUMB-1 was tagged with HA sequence (pAd-HA-NUMB1), and the protein of Numb-1 wih HA was detected by immunoblots between 72 and 95KD. (B) Immunoblots showing the expression of various cell-cycle markers in control and Numb-1 overexpressing cell lines. Increased Numb-1 expression upregulated cyclin B1, cdc2 and p21 levels but did not affect cyclin D1. (C) Increased Numb-1 expression upregulated cyclin B1 and cdc2 in vivo. Immunohistochemistry (IHC) was performed in xenograft samples from mice with Ad-Null and Ad-NUMB-1 tumor. IHC showed higher expression of cyclinB1 and cdc2 in the cytoplasma and nucleus in Numb-1 overexpression samples. (D) Overexpression of Numb-1 in KYSE30 cells did not affect Aurora-A expression but significantly suppressed its activity, as indicated by the decrease in p-Aurora-A, possibly leading to G2/M arrest consistent with previous studies. (E) Lysates of KYSE30 cells were immunoprecipitated (IP) with either preimmune IgG (negative control) or anti-Aurora A, anti-p53 (positive control), or anti-NUMB-1 antibodies followed by immunoblotting (IB) with the antibodies indicated to show co-IP of Aurora-A and Numb-1.

As shown in previous literatures, NUMB was identified as an inhibitor of NOTCH signaling [16, 17]. In our system, we performed experiments to investigate the regulation of NOTCH pathway by overexpression of NUMB-1. The results showed that the expression of NICD (an intracellular domain of Notch) as well as its downstream protein Hes-1 were inhibited by overexpression of NUMB-1 (Fig. 5A). Of interest, in order to clarify whether NUMB-1 arrests ESCC cells specifically in G2 phase or in M phase, various cell cycle check points were tested. Western blot analysis revealed that Numb-1 overexpression upregulated cyclin B1, cdc2, p53, and p21 levels but had no effect on cyclin D1 in KYSE150 cells (Fig. 5B). In addition, we have performed extra experiments to detect the change of cyclin B1 and cdc2 in xenografts from animal experiments by immunohistochemistry assays, and found that the expression of cyclin B1 and cdc2 were both upregulated in Ad-NUMB1 tissues camparing with the Ad-Null tissue(Fig. 5C), which is consistent with our in vitro observations. Furthermore, overexpression of NUMB-1 decreased the phosphorylation of Aurora A (T288), which is believed to be the active form of Aurora A (Fig. 5D). We further elucidated the molecular mechanism of NUMB-1-mediated cell cycle arrest by performing co-immunoprecipitation (co-IP) experiments and found that, in addition to its known partner p53, NUMB-1 also interacted with Aurora-A, a factor important to centrosome maturation and crucial for G2 to M phase transition (Fig. 5E). In order to test whether the interaction between Aurora A and NUMB-1 is p53 dependent, we utilized siRNA to knockdown p53 expression. As shown in supplemental Fig. S2, we successfully knockdown over 80% of endogenous p53, and still were able to detect interaction between Aurora A and NUMB-1. Thus, these data suggest that NUMB-1inhibits notch pathway and induces cell cycle arrest via inhibiting Aurora A activity. The functional interaction between NUMB-1 and Aurora A might be p53 independent.

### NUMB-1 overexpression doesn't impact drug sensitivity of cisplatin and 5-Fu

We evaluated drug sensitivity by MTT test in KYSE150 cells with or without overexpression of NUMB-1 after treating them with concentration gradients of cisplatin and 5-FU which are often used as chemotherapy regimen for ESCC patients. However, no significant difference was found between cells with or without overexpression of NUMB-1, and the cell survival curves were showed in supplemental fig. S3.

## DISCUSSION

Recent studies have identified NUMB as a tumor suppressor [26] that is frequently downregulated in breast cancer and non-small cell lung cancer [27], however, little is known about the clinicopathological significance of NUMB in ESCC. Interestingly, our preliminary immunohistochemistry (IHC) data indicate high expression of NUMB is associated with poor prognosis suggesting that NUMB functions as an oncogene in ESCC; moreover, expression of NUMB mRNA in the ESCC tissues was found to be isoform specific. So far, information on the specific NUMB isoforms accounting for these functional differences in various tissues has been lacking. In the current study, we examined the correlation between NUMB-1 expression and patient prognosis in ESCC patients. We found decreased NUMB-1 mRNA levels in 66.7% of primary ESCC patients, and low expression of NUMB-1 mRNA was associated with shorter EFS (*p*=0.029, HR: 2.088, 95% CI: 1.078-4.045). Furthermore, downregulation of NUMB-1 mRNA was the only significant prognostic factor in this ESCC cohort as determined by multivariate analysis (*p*=0.037, HR: 2.142, 95% CI: 1.047-4.381). Taken together, *in vitro* and *in vivo* data demonstrated that NUMB-1 plays a tumor suppressive role in ESCC.

Our results showed that a decrease in NUMB-1 was linked to advanced tumor stage in ESCC patients (*p*=0.048, χ^2^ test). Since tumor growth, metastasis, and recurrence influence tumor stage and are impacted by cell proliferation and induction of EMT [28], we evaluated the effect of NUMB-1 on these processes in ESCC cells. We discovered that NUMB-1 suppressed FBS-induced migration and invasion by preventing EMT. As shown in previous literatures, Numb is an inhibitor of Notch signal pathway in Drosophilia [6]. In mammalian, Numb proteins promote Notch1 receptor ubiquitination and degradation of the Notch1 intracellulardomain (NICD) [44]. However, the interaction of NUMB isoforms and Notch pathway is still unclear. In our study, overexpression of NUMB-1 down-regulated the expression of NICD and its downstream protein Hes-1 (Fig. 5A) which is consistent with the regulation of Notch Pathway by NUMB. More interestingly, we found NUMB-1 inhibited cell proliferation by inducing cell cycle arrest with various evidence. Cyclin B1, cdc2, and p21 (cell-cycle related proteins) were all upregulated and an increase in aneuploidy was observed upon ectopic expression of NUMB-1. Studies from the literature have shown that high expression of cyclin B1-cdc2 only occurs in the mitotic (M) phase, not the G2 phase [29], and that mitotic entry and exit are controlled by cyclin B1-cdc2 activation and inactivation, respectively [30]. Based on these studies, our results on cyclin B1 and cdc2 suggest that cell cycle progression is arrested in M phase by NUMB-1. This is consistent with a previous study in which overexpression of NUMB by deletion of Musashi, a known repressor of NUMB translation, resulted in glioma cells arresting in M phase [31, 32]. In contrast, a recent study in human melanoma cells demonstrated that a targeted knockdown of NUMB expression disrupts its ability to interact with the serine/threonine polo-like kinase Plk1 thereby causing G2–M arrest and reducing cell growth[33]. The reason for the functional disparity of NUMB, where both overexpression and downregulation of NUMB result in G2/M phase arrest, is still unclear. It is possible that the biologic effect of NUMB is isoform specific, as supported by a recent study which found that alternative splicing of NUMB produces functionally distinct protein isoforms that influence the proliferation of lung cancer cells differently [34]. Further studies need to be performed in order to determine the distinct role of different NUMB isoforms in ESCC.

Aurora-A is a Ser/Thr kinase involved in the separation of centrosomes and in spindle assembly during mitosis [35]. Interestingly, we found that cell-cycle distribution following NUMB-1 overexpression closely resembled that seen upon inhibition of Aurora-A activity [36]. Furthermore, studies have shown that Aurora-A is amplified in ESCC and plays an important role in carcinogenesis, inhibition of apoptosis by upregulation of Bcl-2 expression and metastasis, and upregulation of MMP-2 [37-39]. Our study provided the first evidence that Numb-1 binds to Aurora-A and decreases Aurora-A T-288 phosphorylation in ESCC cells. The phosphorylated form of Aurora-A is recognized as the active form in tumors [40], and downregulation of its activity/phosphorylation contributed to prolonged centrosome maturation [41, 42], M phase arrest, and an increase in aneuploidy. Interestingly, in *Drosophila* neural precursor cells, activation of Aurora-A was shown to trigger phosphorylation of Numb and was responsible for the asymmetric localization of Numb during mitosis [43]. Therefore, to clarify the role of NUMB isoforms in ESCC, we need to further delineate the mechanism of regulation among Aurora A, NUMB-1, and the other NUMB isoforms.

The interaction between NUMB and p53 has been well studied [14, 15]. However, in our study, we found NUMB-1 co-IPed with Aurora A, is it possible that the binding of NUMB-1 to Aurora A is mediated by p53? To answer this question, we have established a p53 knockdown model in KYSE150 cell line by siRNA against p53 and retested the interaction of NUMB-1 and Aurora A. The results showed that NUMB-1 was still able to interact with Aurora A. It suggested that the binding of NUMB to Aurora A might be p53 independent. However, since siRNA cannot totally ablate expression of p53, further experiment with CRISPR-mediated p53 knockout cell line is our ongoing project to demonstrate the function of p53 in interaction between NUMB-1 and Aurora A. Of interest, we also found overexpression of NUMB-1 inhibits tumor growth in KYSE30 cell line and KYSE150 cell line, harboring mutant and wild type p53 respectively, which suggested the tumor suppressor function for NUMB-1 is p53 independent. Nevertheless, the underlying mechanisms for the functional interaction among p53, NUMB-1and Aorora A need to be further studied.

Given that the NUMB protein family is a complex system with distinct structures and functions, clearly, there are some questions required future investigations. For example, paracrine effects have been shown to play a role in tumor progression [45, 46], which has not been linked to NUMB signaling pathway. To answer the question whether NUMB-1 plays a role in paracrine effects in tumor growth, in vitro co-culture of cells with or without overexpression of NUMB-1, and the animal models with NUMB-1 overexpressing tumor and control tumor were xenografted into the same mouse (used in current study), and with these two tumors xenografted in different mice (ongoing projects) might provide insights into this question. Furthermore, in order to design PCR primer sets that are specific for NUMB-1, we had to span the PCR products from different exons of NUMB gene and sequenced the PCR products, then confirmed the isoform specificity for each pair of primer sets, however, the factor that product >950 bp makes real-time PCR impossible. Gene knockdown technology was also difficult in this study due to a lack of specificity for the various NUMB isoforms. To date, there is no antibody specific for NUMB-1. Therefore, in our study, in order to test the tumor suppressor roles for the NUMB-1, we have used adenovirus encoding NUMB-1 with a hemagglutinin (HA) tag (pAd-HA-NUMB1). Thus, we can detect HA-NUMB-1 by the molecular weight shifting (higher band in Fig. 5D) or anti-HA antibody (Fig. 5A). In addition, one of the current efforts in our laboratory is generating a NUMB-1 specific antibody. These challenges need to be overcomed in the future.

Our data showed, for the first time, that NUMB-1 can serve as a biomarker for prognosis in patients and as a tumor suppressor to halt progression of ESCC. Specifically, downregulation of NUMB-1 is associated with poor prognosis while overexpression of NUMB-1 results in G2/M arrest by inhibiting Aurora A T-288 phosphorylation. Further investigation of the biological function of other NUMB isoforms and their mechanism of regulation in human patients with ESCC will not only facilitate our understanding of ESCC development and progression but will also provide potential therapeutic targets for ESCC treatment.

## MATERIALS AND METHODS

### Patients and follow-up

The study population consisted of 75 Chinese ESCC patients (median age, 60 years; range, 36-79 years) who underwent radical surgery, between January 2009 to December 2011, at either the First Affiliate Hospital (50 cases) or the Cancer Center (25 cases) of Sun Yat-sen University. None of the patients received neoadjuvant therapy before surgery. Pathology was re-reviewed to confirm histology and tumor grade by at least two pathologists, and all cases of ESCC were staged according to the American Joint Committee on Cancer (AJCC) 7th edition, 2010 [29]. There were 5 cases of stage I, 40 cases of stage II, 28 cases of stage III, and 2 cases of stage IV. Follow-up care after surgery, including radiographic imaging and histologic verification of recurrence, was performed at the follow-up center in the two hospitals. The period of follow-up care ranged from 6 months to 59 months with a mean of 37 months. The clinical features of these cases are shown in Table 1. All protocols, regarding human subjects, were approved by the Ethics Committee and Institutional Review Board of both the First Affiliated Hospital and the Cancer Center of Sun Yat-sen University. A written consent form was procured from each participant before the study.

### RNA preparation and reverse transcription PCR

75 pairs of primary ESCC tumors and matched adjacent normal tissues from the proximal resection margins were collected and stored in the liquid nitrogen tank for total RNA extraction. Total RNA was extracted using Trizol reagent (Invitrogen, Cat. # 15596-026) and cDNA was generated from 2μg of total RNA with oligo dT primers and reverse transcriptase (Thermo, Cat. # K1622), according to the manufacturer's instructions. The cDNA product was used as a template for subsequent PCR of NUMB-1 using sequence-specific primers designed to amplify cDNA regions. One tenth of the prepared cDNA was used in PCR reactions containing 0.2μmol/L of each primer (forward and reverse), 100μmol/L deoxynucleotide triphosphates (dNTPs), 1.5 mmol/L MgCl2, and 0.02 unit/μL Taq DNA polymerase (Thermo, Cat. # K1081). Since our discovery is specific for NUMB-1, it is most important to differentiate NUMB-1 from other isoforms. In briefly, we designed the specific primers for specific NUMB isoform 1 as primer 1(span exon5 and exon 6 boundaries) and primer 2 (span exon12 and exon 13 boundaries).

Primers for NUMB isoform 1 are as follows:

Forward:5'-GAAAGGAAGTTCTTCAAAGGC-3'; Reverse:5'-CTAGAGCAGTATGGGCTGG-3'

Primers for GAPDH are as follows:

Forward:5′-GAGTCAACGGATTTGGTCGT-3′;

Reverse:5′-GATCTCGCTCCTGGAAGATG-3′

### Adenoviral construct for overexpressing NUMB-1

Virus construct was generated as previously reported [47]. Briefly, The E1/E3-deficient, recombinant adenovirus serotype 5 was tagged with hemagglutinin (HA) sequence, and a cytomegalovirus (CMV) promoter driving a mouse NUMB-1 cDNA fragment encoding NUMB-1 was inserted. This was constructed and generated by SinoGenoMax Co.Ltd (www.sinogenomax.com/en/). And the Ad-CMV-HA was used as control.

### siRNA Transfection

Oligonucleotide siRNA duplex was synthesized by Shanghai Gene Pharma (Shanghai, China). The sequence of p53 siRNA was: 5'-CUACUUCCUGAAAACAACGTT-3' for siRNA 1; 5'-UGGUUCACUGAAGACCCAGTT-3' for siRNA 2; 5'-GACUCCAGUGGUAAUCUACTT-3' for siRNA 3; the sequence of negative control was 5'-UUCUCCGAACGUGUCACGUTT-3'. The transfection of siRNA in KYSE150 cells was carried out with Lipofectamine 2000 (Invitrogen) according to the manufacturer's instruction.

### Cell culture and animals

ESCC cell lines (KYSE150, KYSE30, KYSE180, and TE-1) were provided by Professors Guan XY, which were obtained from DSMZ, the German Resource Center for Biological Material[48], and cultured in RPMI-1640 (Invitrogen, USA) or DMEM supplemented with 10% FBS in a humidified incubator at 37°C, 5% CO2. Female nude BALB/c mice, weighing 18-22g, were bred in the animal facility of Sun Yat-sen University. Animal care and experiments were approved and performed in compliance with the guidelines for the Welfare of Experimental Animals in Sun Yat-sen University. 2×10^6^ cells were subcutaneously injected into the left and right flanks of 6-week-old female nude BALB/c mice (n=5 per NUMB-1 group and Null group). Tumors were measured with calipers every 3 days for 20 days after initial detection, and tumor volume in mm^3^ was calculated by the formula: Volume = (width)^2^ × length × 3.14/6 [49]. Following sacrifice of the animals, tumors were excised, weighed, and photographed.

### Wound healing assay

Cells were seeded in six-well plates and cultured under permissive conditions until 90% confluence was reached. After starving the cells for 24 h in medium without EGF or FBS, the confluent cell monolayer was lightly and quickly scratched with a pipette tip in a straight line. The debris was removed and the edge of the scratch was smoothed with PBS washing. The wound healing assays were performed in growth factor-free medium in order to exclude any proliferative differences. The gap was then inspected and photographed, at a 200× magnification by phase contrast microscopy, at the indicated times shown in the Figures. A minimum of three independent experiments was performed.

### Boyden chamber assay

Cells were seeded in growth factor-free medium (no EGF or FBS) in the top chamber (Corning, USA), which coated 20% Matrigel (BD #354234) on up-surface, while medium containing EGF or FBS was added to the bottom chamber. After 24 h, the cells on the undersurface of the chambers were fixed with 1% paraformaldehyde and stained with hematoxylin. The number of cells in ten random fields of view, at 400× magnification, were counted for each filter. Three independent experiments were performed, and the data are presented as the mean ± SD.

### Cell proliferation assay

Cell growth was measured by both colony formation assay and 3-(4,5-dimethylthiazol-2-yl)-2, 5-diphenyl-tetrazo-lium bromide (MTT) assay. For the colony formation assay, cells were plated into 6-well plates, allowed to grow for 14 days, fixed and stained with crystal violet, and then counted. MTT assay was performed as described previously [49]. Infected cells were plated at 1000 cells per well in 96-well flat-bottom plates. At 1, 2, 3, 4, and 5 days following plating, cells were stained with 20 μL sterile MTT dye (0.5 mg/ml, Amesco) for 4 h in the incubator. The culture medium was then removed, 120 μL of DMSO was added, and the absorbance value was measured on a microplate reader (Bio-Rad, USA) at 570nm.

### Cell cycle analysis

Cells were harvested, washed twice with ice-cold PBS, and fixed with 70% ethanol for 12 h or overnight at 4 °C. Cells were washed twice using ice-cold PBS and then incubated with propidium iodide (50 μg/mL) and RNase (50 μg/mL) for 30 min at room temperature in the dark. DNA content was analyzed by flow cytometry (Beckman Coulter, Cytomics FC500, Fullerton, CA).

### Western blot analysis and co-immunoprecipitation

Protein extractions and western blot assays were conducted as described previously [49]. Membranes were incubated with indicated primary antibodies for Numb (Anti-Numb polyclonal antibody was generated by Abmart, and affinity purified as previously described. [50] (1:2000), E-cadherin (1:200, Santa Cruz Biotechnology, USA), Vimentin (1:50, Millipore, USA), α-SMA (1:1000, abcam, USA), p21^WAF1/CIP1^ (1:1000, Cell Signaling Technology, USA), p53(1:2000, Cell Signaling Technology, USA), cyclin D1 (1:1000, Cell Signaling Technology, USA), cyclin B1 (1:1000, Cell Signaling Technology, USA), cdc2 (1:1000, Cell Signaling Technology, USA), PARP (1:200, Cell Signaling Technology, USA), Caspase-3 (1:1000, Cell Signaling Technology, USA), Aurora-A (1:1000, bioworld, USA), or p-Aurora-A (1:100, Cell Signaling), Notch-1(NICD) (1:500, Cell Signaling Technology, USA), Hes-1 (1:1000, Cell Signaling Technology, USA) at 4°C overnight, followed by incubation with horseradish peroxidase-conjugated secondary antibodies, and detected by chemiluminescence (Bio-Rad, USA). For immunoprecipitation, 1 mg total protein was incubated with 2μg antibody in lysis buffer at 4ºC overnight. The antibody was pulled down by incubating the lysates for 2h with 50 μL Protein A/G beads (Santa Cruz Biotechnology, USA). Beads were washed 4 times with RIPA buffer (5min/wash) and collected by centrifugation at 3500g for 10 min. The beads were resuspended in 2× loading dye, boiled for 5 min, and followed by western blot analysis.

### Immunohistochemical (IHC) Analysis

IHC was performed as previously described[49]. Formalin-fixed xenografts tissue was imbedded in paraffin and cut by microtome into tissue sections (4-μm thick) for immunostaining according to standard techniques. The primary antibody was cyclin B1 (1:100, Bioworld Technology, USA), cdc2 (1:100, Bioworld Technology, USA). The negative control was performed for each specimen by omitting the primary antibody in the procedure. Subjective assessments of the intensity of staining were performed according to the manufacturer's package insert.

### Time-lapse imaging

Time lapse imaging was performed as described previously[51]. To image chromosome dynamics during the cell cycle, KYSE150 cells was firstly incubation with Hoechst 33342 at a concentration of 0.01μg/ml that would not harmful for cells[51], then washed twice by PBS and cultured in chambers and imaged at 37°C on the microscope stage. The stage is environmentally controlled for temperature (37°C) and atmosphere (humidified, 5%CO2), allowing cells to grow normally. Time-lapse fluorescence images were obtained using excitation from a filtered metal halide lamp in the Nikon TE2000S microscope. The filter used for Hoechst 33342 excitation had a peak at 365±20 nm. Fluorescence was detected at 460±25 nm. The exposure time for collection of Hoechst 33342 fluorescence was constant at 0.6s per image scan. Images of cells for each treatment condition were taken with a 10× objective every 30 min for up to 36 hours. At least three independent experiments were carried out for each experimental condition.

### Statistical analysis

Continuous variables are shown as mean ± standard deviation (SD), and categorical quantitative data are presented as counts and proportions. The clinical characteristics and follow-up data were analyzed by Student's t test as a parametric test and Mann-Whitney U test as a non-parametric test; ratios of categorical variables were compared by Chi-square test. Univariate analysis and multivariate Cox regression analysis were performed to determine the impact of various clinical characteristics on the outcomes of ESCC patients. EFS is defined as the length of survival time from the time point of surgical treatment to the time point that the tumor metastasis or recurrence is confirmed in our cohort. EFS and OS were evaluated by Kaplan-Meier analysis. Statistical significance was set at *p*<0.05. Statistical analysis was performed with SPSS 18.0 software (SPSS, Chicago, IL).

## SUPPLEMENTARY MATERIAL AND FIGURES


