# Supplementary material for: The tumor suppressive role of NUMB isoform 1 in esophageal squamous cell carcinoma
Source: Oncotarget. 2014 Jun 26;5(14):5602–14. doi: 10.18632/oncotarget.2136 (PMC4170621; doi:10.18632/oncotarget.2136)
Supplement: Supplementary file 1 [file oncotarget-05-5602-s001.pdf]

The tumor suppressive role of NUMB isoform 1 in esophageal squamous cell carcinoma

Supplementary Material

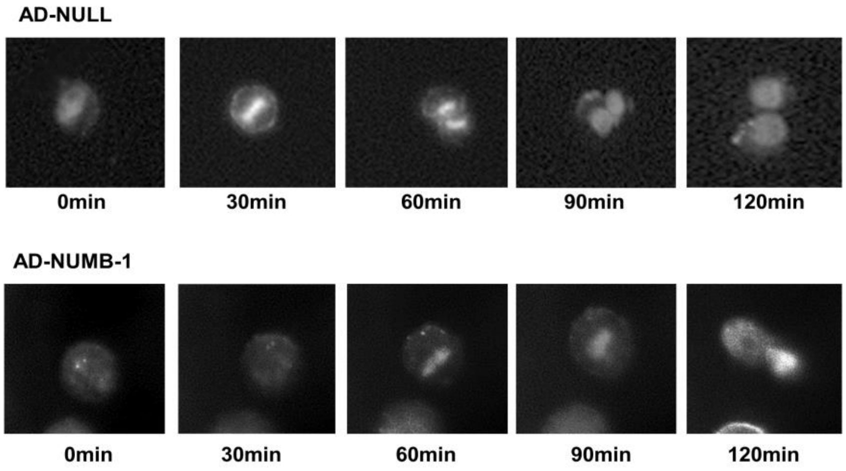

Supplemental Fig. S1: Dynamics of NUMB-1 induced cell cycle arrest in G2/M phase by the time-lapse imaging technology. Time-lapse imaging was performed to observe dynamically the inhibition of cell cycle arrest by NUMB-1 overexpression. KYSE150 cells transfected with Ad-Null and Ad-NUMB-1 were incubated with Hoechst 33342 at a concentration of 0.01μg/ml. The chromosome dynamics during the cell cycle were recorded by fluorescence microscope every 30 minutes. Representative images of chromosome dynamics of cells were showed (Top, Ad-Null cells; bottom, Ad-NUMB-1 cells), and the cell cycle arrest in G2/M phase was observed in Ad-NUMB-1 cells.

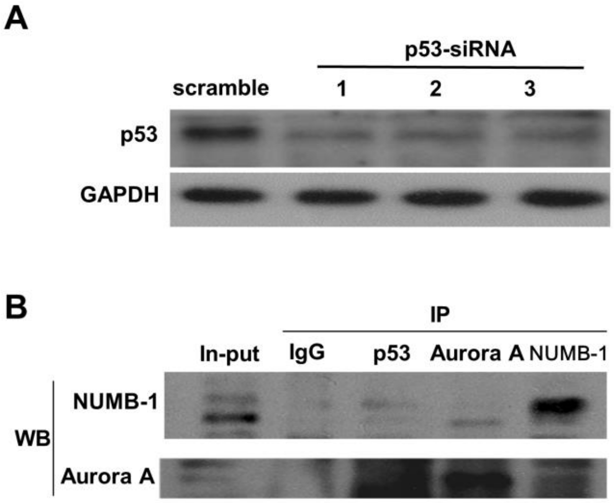

Supplemental Fig. S2: Co- immunoprecipitate (co-IP) of Aurora-A and Numb-1 in p53 knockdown KYSE150. (A) p53 was knockdown by siRNA efficiently. (B) Lysates of p53 knockdown KYSE150 cells were immunoprecipitated (IP) with either preimmune IgG (negative control) or anti-Aurora A, anti-p53 (positive control), or anti-Numb-1 antibodies followed by immunoblotting (IB) with the antibodies indicated , the results showed Numb-1 interacted with Aurora A in p53 knockdown KYSE150 cells.

A

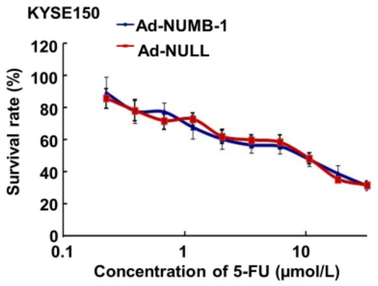

B

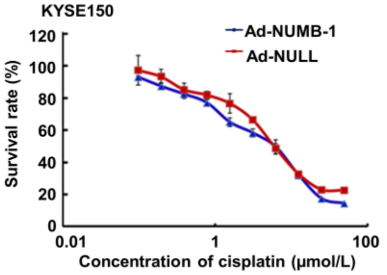

Supplemental Fig. S3: The cytotoxicity of 5-FU and cisplatin in Ad-Null and Ad-NUMB-1 KYSE150 cells. The cytotoxicity of 5-FU and cisplatin was performed by MTT assay, and there was no significant difference between Ad-Null and Ad-NUMB-1 KYSE150 cells. (a)The IC50 values of 5-FU was 6.274, 6.300 μmol/L for Ad-Null and Ad-NUMB-1 KYSE150 cells, respectively. (b) The IC50 of cisplatin was 6.170, 5.648 μmol/L for Ad-Null and Ad-NUMB-1 KYSE150 cells, respectively.
